# Efficacy and cerebral mechanism of acupuncture and moxibustion for treating primary dysmenorrhea: study protocol for a randomized controlled clinical trial

**DOI:** 10.1186/s13063-022-06675-1

**Published:** 2022-11-28

**Authors:** Xiaohui Dong, Jie Yang, Wei Wei, Ling Chen, Menghua Su, Aijia Li, Xiaoli Guo, Liying Liu, Shenghong Li, Siyi Yu, Fang Zeng

**Affiliations:** 1 Acupuncture and Tuina School/The 3rd Teaching Hospital, Chengdu University of Traditional Chinese Medicine, Chengdu, Sichuan China; 2 Acupuncture and Brain Science Research Center, Chengdu University of Traditional Chinese Medicine, Chengdu, Sichuan China; 3 State Key Laboratory of Southwestern Chinese Medicine Resources, and Innovative Institute of Chinese Medicine and Pharmacy, Chengdu University of Traditional Chinese Medicine, Chengdu, Sichuan China

**Keywords:** Acupuncture, Moxibustion, Primary dysmenorrhea, Functional magnetic resonance imaging, Central mechanism

## Abstract

**Background:**

Acupuncture or moxibustion has been proven to be effective for patients with primary dysmenorrhea (PDM). However, the respective advantages and potential central mechanism of acupuncture and moxibustion are worthy of investigating to promote their further application.

**Methods:**

In this randomized controlled neuroimaging trial, 72 patients with PDM will be randomly assigned to three groups: acupuncture treatment group, moxibustion treatment group, and waiting list group. The acupuncture treatment group and moxibustion treatment group will receive acupuncture or moxibustion, respectively, for a total of 3 sessions over 3 consecutive menstrual cycles, and the waiting list group will not take acupuncture or moxibustion during these 3 menstrual cycles. The COX Menstrual Symptom Scale (CMSS), visual analog scale (VAS), and Pain Catastrophizing Scale (PCS) will be used to evaluate the clinical efficacy. The Self-rating Depression Scale (SDS), Self-rating Anxiety Scale (SAS), and 36-Item Short Form Health Survey (SF-36) will be used to assess the mental state and quality of life at baseline and at the end of treatment. Functional magnetic resonance imaging (fMRI) will be performed for detecting the cerebral activity changes at baseline and at the end of the treatment. The clinical data and imaging data will be analyzed among the groups. Correlation analysis will be conducted to investigate the relationship between brain functional changes and symptom improvement.

**Discussion:**

The application of the randomized controlled neuroimaging trial will provide objective and valid evidence about how acupuncture and moxibustion treatment relieve menstrual pain. The results of this study would be useful to confirm the potential similarities and differences between acupuncture and moxibustion in clinical efficacy and central mechanism for patients with PDM.

**Trial registration:**

Chinese Clinical Trial Registry ChiCTR2100043732. Registered on 27 February 2021

## Background

Primary dysmenorrhea (PDM) is one of the most common gynecological disorders with a prevalence of around 45–95% worldwidely [1]. It is characterized by recurrent, crampy, lower abdominal pain during menstruation in the absence of organic pelvic diseases [2, 3]. In some severe cases, patients may suffer from other symptoms including nausea, vomiting, limb cold, sweating, and even fainting accompanied by lower abdominal pain [4]. PDM significantly impacts the patients’ quality of life (QoL) and leads to high rates of school or work abence [5–8]. The current medical treatments for PDM range from nonsteroidal anti-inflammatory drugs (NSAIDs) to hormonal therapy [3, 7]. However, the side effects and unsatisfactory efficacy limit their clinical application [9–11]. Therefore, more and more doctors and patients are inclined to choose complementary alternative therapies.

Acupuncture and moxibustion are critical components of complementary alternative therapies and have good therapeutic effects on PDM [12, 13]. Acupuncture and moxibustion pertain to external therapies of traditional Chinese medicine (TCM). They are both guided by the traditional Chinese acupoint and meridian theory and functions in dredging the meridians and promoting the *qi* and blood circulation to treat diseases, although their procedures and manipulations are different. Acupuncture is to insert a needle into acupoints and impose a certain manipulation to achieve the purpose of treatment, while moxibustion is to place a burning mugwort directly or indirectly on acupoints to treat diseases. A number of studies had shown that whether acupuncture or moxibustion alone or a combination of both can relieve pain effectively to treat PDM [14–16]. Our previous studies have also verified the efficacy of moxibustion in alleviating menstrual pain and improving its related symptoms [13, 15]. In addition, previous studies have shown that the integration of the central system to acupuncture or moxibustion stimulation plays a crucial role in exerting the therapeutic effect [17, 18]. For example, researchers have found that both acupuncture and moxibustion improve cortex-subcortical coupling in remissive CD patients but through relatively different modulatory pattern [19]. Thus, although acupuncture and moxibustion are both effective in treating PDM, their respective advantages in effect and the underly mechanism remain unclear and need to be further investigated.

Therefore, we designed this randomized controlled neuroimaging trial aiming to (1) compare the therapeutic effects of acupuncture and moxibustion alone treating for PDM so as to investigate their respective advantages, (2) investigate the potential central mechanism of acupuncture and moxibustion for PDM using functional magnetic resonance imaging (fMRI), and (3) explore the potential correlations between cerebral activity changes and the improvement of clinical variables elicited by acupuncture or moxibustion.

## Methods/design

**Fig. 1:**
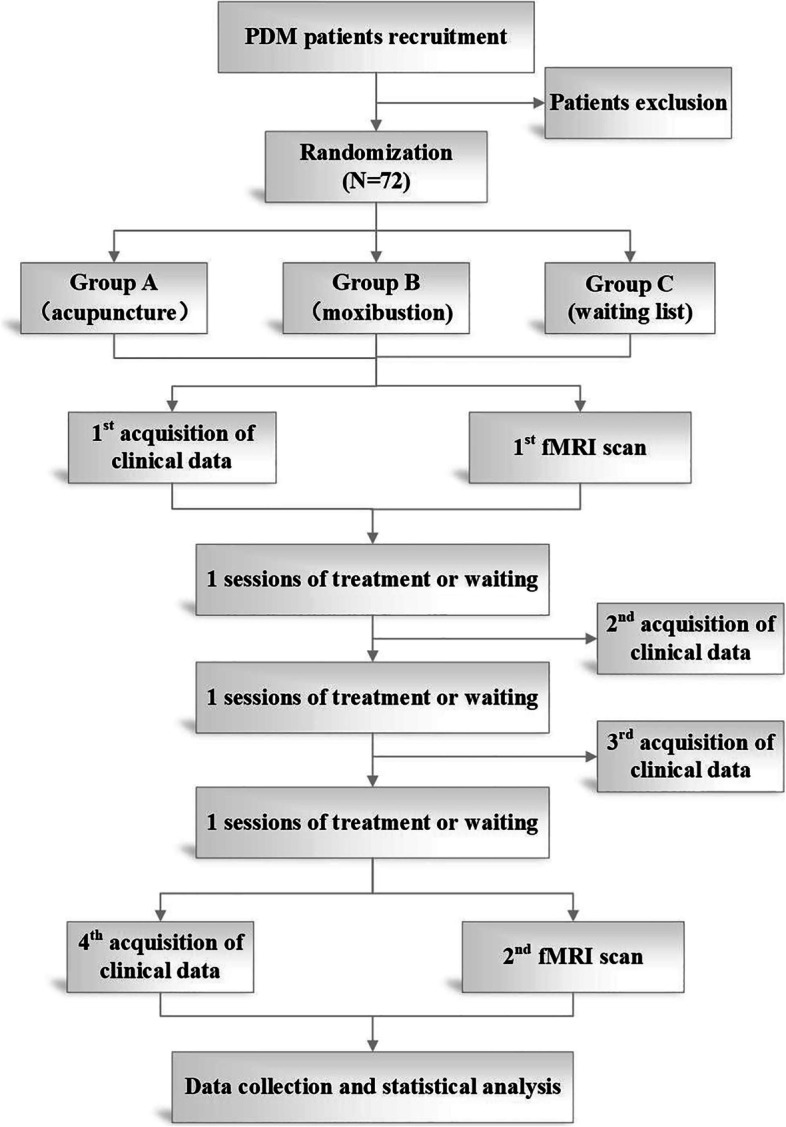
Flowchart of the study. A total of 72 eligible patients with PDM will be randomly assigned to three groups: acupuncture treatment group, moxibustion treatment group, and waiting list group. Patients in the acupuncture treatment group and moxibustion treatment group will receive acupuncture or moxibustion, respectively, for a total of 3 sessions over 3 consecutive menstrual cycles, and the waiting list group will not take acupuncture or moxibustion during these 3 menstrual cycles. Both the outcome assessments and fMRI scan will be performed at baseline and at the end of treatment. The central mechanism of acupuncture and moxibustion in the treatment of PDM will be analyzed after data collection. fMRI, functional magnetic resonance imaging; PDM, primary dysmenorrhea

This is a randomized controlled paralleled neuroimaging trial. A total of 72 patients meeting the inclusion criteria will be recruited and randomly divided into three groups (the acupuncture treatment group, the moxibustion treatment group, and the waiting list group) by the ratio of 1:1:1 after a 3-month baseline period. Acupuncture and moxibustion treatment period will last for 3 menstrual cycles, while the waiting list group will not take acupuncture or moxibustion treatment during these 3 menstrual cycles. All patients will be allowed to use analgetic (VAS ≥ 8 cm), and the name, dose, and exact time of the medication will be recorded in the dysmenorrhea diary. Clinical outcome measurements and fMRI scan will be conducted at baseline and at the end of treatment. The study procedures are detailed in Fig. 1.

The study protocol conforms to the SPIRIT 2013 statement (Standard Protocol Items: Recommendations for Interventional Trials) [20]. The protocol has been approved by the Institutional Review Board of the Hospital of Chengdu University of Traditional Chinese Medicine (CDUTCM) (approved number: 2021KL-010) and registered at Clinical Trial Registry (registration number: ChiCTR2100043732).

### Participants and recruitment strategy

Patients will be recruited from the outpatient clinic in the Gynecology Department of the Affiliated Hospital of CDUTCM and the campus of CDUTCM. The recruitment strategy mainly includes posting advertisement, delivering leaflets, and distributing information on WeChat (the largest social media platform in China). All patients will be informed of the study procedures, potential benefits, and risks, and written informed consent will be obtained before the allocation.

### Inclusion criteria

Patients fulfilling the following criteria will be included: (1) nulliparous women aged 18–30 years old, (2) right-handed, (3) meeting the diagnostic criteria of PDM under the Primary Dysmenorrhea Consensus Guideline [21], (4) having regular menstrual cycles (28 days ±7 days), (5) having an average score of the menstrual pain visual analog scale (VAS) of at least 4 cm (range of 0 to 10 cm) during the last menstrual period, and (6) having signed informed consent form.

### Exclusion criteria

Patients matching any of the following criteria will be excluded: (1) having been diagnosed with secondary dysmenorrhea caused by polycystic ovarian syndrome, endometriosis, uterine myoma, or other gynecological problems confirmed by B-ultrasound and gynecological examination, (2) having MRI contraindications such as severe claustrophobia, pacemakers, or implanted ferromagnetic metal, (3) experiencing severe depressive or anxiety disorder, (4) complicating with life-threatening diseases such as psychiatric, neurologic, cardiovascular, cerebrovascular, liver, kidney, and hematopoietic system illnesses, (5) being pregnant or lactating or preparing to be pregnant, (6) using analgesics and sedatives 2 weeks prior to enrollment, and (7) participating in other clinical trials.

### Sample size

According to the statistical requirements of neuroimaging studies, 12–15 individuals in each group is the reasonable sample size for stable cerebral responses [22, 23]. Considering a drop-out rate of 20% and possible excessive head motion during scanning, a sample size of 24 in each group is required, and a total of 72 PDM patients will be recruited.

### Randomization and blinding

Randomization will be implemented using computer-generated randomization digital table which is created by Excel’s rand function. Seventy-two eligible PDM patients will be randomly assigned to the acupuncture group, moxibustion group, and waiting list group (1:1:1 ratio). Random information will be put into an opaque, sealed envelope and saved by those who will not participate in the study. Due to the particularity operation of acupuncture and moxibustion, it is difficult to blind operators and patients. However, outcome assessors and statisticians were blinded to the procedure and the result of randomization, group allocation, and intervention to reduce the risk of bias.

### Interventions

#### Acupuncture interventions

Patients in this group will receive manual acupuncture at CV4 (Guan yuan) and SP6 (San yinjiao) with disposable sterile filiform needles (0.25 × 40 mm, Huatuo Medical Instrument Co., Ltd., China). Based on our previous studies and academic literature data mining, these acupoints have been proven to be effective and most frequently used for PDM [13, 24, 25]. Needles will be perpendicularly inserted into the acupoints at a depth of 20–30 mm after skin disinfection using alcohol; acupuncturists will then bi-directionally twist the needles by 90–180°, lifting and thrusting the needles with the amplitude of 3–5 mm for 1–1.5 Hz to induce *Deqi* sensation. After the *Deqi* sensation is attained, the needles will be retained at the acupoints for 30 min.

#### Moxibustion treatment

Patients in this group will receive mild moxibustion at CV4 and SP6 with moxa sticks (Z32021062, Oriental Moxa Co., Suzhou, China). The moxa sticks are made of moxa floss and are cylindrical, with a diameter of 1.5 cm and a length of 20 cm. The ignited moxa sticks will be applied approximately 2–3 cm above the dermal layer of the acupoints to produce a mildly warm and comfortable sensation that is similar to a *Deqi* sensation in acupuncture. Moxibustion at each point commonly lasts for about 10–15 min [13].

Patients will receive acupuncture or moxibustion treatment 5 days before the onset of menstruation, once a day, 5 days a session for a total of 3 sessions over 3 consecutive menstrual cycles. All the acupuncture and moxibustion manipulation will be performed by two licensed acupuncturists with at least 3 years of clinical experience.

### Waiting list group

Patients in the waiting list group will not receive acupuncture or moxibustion intervention and only need to maintain their previous habits. The participants will also complete the examination and evaluation at the corresponding time point. Considering the ethical requirements, all patients in this group will receive free acupuncture or moxibustion treatment at the end of the trial.

### Measurements

The measurements mainly include basic information collection, symptom measurement, and neuroimaging scanning assessments. All measurements will be performed independently by trained assessors. The study schedule is exhibited in Table 1.

**Table 1:** Study schedule of enrollment, intervention, and assessments

	Baseline	Allocation	Treatment		
Time point	− 3 months	0 months	1 month	2 months	3 months
**Enrollment**					
Eligibility screen	√				
Informed consent	√				
Demographics	√				
Medical history	√				
Physical examination		√			
Randomization		√			
**Interventions**					
Group A (acupuncture)			√	√	√
Group B (moxibustion)			√	√	√
Group C (waiting list)			√	√	√
**Assessments**					
MRI scans		√			√
VAS		√	√	√	√
CMSS		√	√	√	√
PCS		√	√	√	√
SAS		√			√
SDS		√			√
SF-36		√			√
**Participants safety**					
Adverse events			√	√	√

### Demographic and basic clinical information collection

The demographic information including name, age, height, weight, level of education, and history of PDM and other concomitant diseases will be collected at the baseline. The vital signs including blood pressure, pulse, respiration rate, and temperature will also be measured.

### Symptoms measurement

#### The COX menstrual symptom scale (CMSS)

The CMSS is commonly used to evaluate dysmenorrhea symptoms and other accompanying symptoms [26]. The scale contains 18 symptom items, each of which includes both total onset time and average severity.

#### Visual analog scale (VAS)

The VAS is a tool widely used to measure pain intensity [27]. Patients will be asked to indicate a perception of pain intensity scored from 1 to 10 (0 = no pain sensation, 10 = the worst pain sensation) along a 100-mm horizontal line.

#### Pain catastrophizing scale (PCS)

The PCS is a 13-item self-report tool used to assess an exaggerated negative orientation towards actual or anticipated pain experiences [28, 29]. The scale measures different features of pain catastrophizing from three subscales: rumination, magnification, and helplessness.

#### Psychological state assessment

The Self-rating Anxiety Scale (SAS) and Self-rating Depression Scale (SDS) will be used to evaluate the emotional state [30, 31].

#### Measurements for the quality of life

The 36-item Short Form Health Survey (SF-36) will be used to evaluate the quality of life [32]. The scale contains 8 dimensions including physical functioning, role limitations due to physical health problems, role limitations due to emotional problems, social functioning, bodily pain, vitality, mental health, and general health.

The VAS, CMSS, and PCS will be measured at baseline and every menstrual cycle throughout the treatment. The SAS, SDS, and SF-36 will be measured at baseline and at the end of the treatment.

#### MRI scan

All participants will receive MRI scans at the baseline and at the end of the treatment. MRI data will be acquired with a 3.0-T magnetic resonance scanner (GE 3.0 T MR750; GE Healthcare, Chicago, IL, USA) at the MRI Center in University of Electronic Science and Technology of China. A comfortable sponge pad is used to fix the subject’s head to minimize head motion, and earplugs are used to attenuate noise. At the same time, all participants will be told to remain motionless, keep their eyes closed, and stay awake during the scanning. The scanning procedure contains a localizer, a high-resolution three-dimensional T1-weighted imaging (3D-T1WI), and a blood oxygenation level-dependent fMRI (BOLD-fMRI).

The scanning parameters will be as follows: 3D-T1WI—repetition time (TR)/echo time (TE) = 2000/30 ms, slice thickness = 1 mm, slice number = 1, matrix size = 128 × 128, and field of view (FOV) = 256 × 256 mm; BOLD-fMRI—TR/TE = 2000/30 ms, flip angle = 90°, slice number = 35, matrix size = 128 × 128, FOV = 240 × 240 mm, slice thickness = 4 mm, and total volume = 240.

### Patient safety

Adverse events (AE) and serious adverse events (SAE) may occur during the acupuncture and moxibustion treatment or neuroimaging scanning. For example, possible adverse events due to acupuncture include subcutaneous hemorrhage, severe pain, and fainting. Possible adverse events due to moxibustion include blisters, redness, itching, and burns. All adverse events will be treated immediately and recorded in the case report form (CRFs) in detail throughout the trial. SAEs will be reported to the Research Ethics Committee within 24 h. Furthermore, all events will be evaluated for their relevance to the intervention and severity.

### Data management and monitoring

Data will be collected by a dedicated researcher, and the quality of the data will be supervised by two independent researchers. The communication between researchers and participants will be strengthened to improve adherence and to promote participant retention. The clinical data will be recorded accurately and timely in printed CRFs. All the neuroimaging data will be stored in dedicated hard drives after every scanning is completed. The Ethics Committee of the First Teaching Hospital of Chengdu University of TCM will be supervising this trial and will make the final decision to terminate the trial. The process will be independent from the investigators and the sponsor. The data safety monitoring board (DSMB) will be established to ensure patient safety and data confidentiality. The DSMB will conduct ongoing safety monitoring and meet every 3 months.

### Data analysis

A detailed statistical analysis plan (SAP) was developed before the initiation of the statistical analysis. The data analysis will be completed by statisticians who are independent from the research team. The SAP includes clinical data analysis and neuroimaging data processing and analysis.

### Clinical data analysis

The Kolmogorov-Smirnov test will be used to test the normal distribution of continuous variables. Continuous variables on normal distribution will be presented by mean ± standard deviation, while continuous variables of skewed distribution were expressed as medians and interquartile ranges (IQRs). Analyses will be performed on an intention-to-treat basis. Multiple imputations will be used to handle the missing data. Paired samples *t*-test will be used to compare the clinical outcomes between baseline and end of treatment in each group. Since there are three groups (acupuncture group, moxibustion group, and waiting-list group) with two time points (pre-treatment and post-treatment), the repeated measures analysis of variance (ANOVA) will be employed to analyze the clinical data. In the 2 × 3 group factorial design, the dependent variables were the clinical data collected from pre-treatment and post-treatment, and data in the three different groups will serve as the independent variable. ANOVA and chi-square test will be used to compare group differences at baseline. ANOVA with Šidák corrections will be used to compare the differences between the groups. Clinical data on skewed distribution will be compared using a non-parametric test. A two-sided test is applied for available data, and a *P* value of less than 0.05 is considered statistically significant. Missing data will be replaced by the data from the latest assessment.

### Neuroimaging data processing and analysis

The fMRI data will be preprocessed and post hoc analyzed by the SPM12 software (http://www.fil.ion.ucl.ac.uk/spm/) and CONN toolbox (http://www.nitrc.org/projects/conn) performed on MATLAB 2015b (MathWorks, Inc., Natick, MA, USA). The preprocessing steps will include slice timing correction, head motion correction, spatial normalization, spatial smoothing, and detrending. After data preprocessing, amplitude of low-frequency fluctuation, regional homogeneity, and functional connectivity will be used to investigate the cerebral responses of the different study groups. ANOVA will be used to evaluate possible cerebral responses in each group by within-group analysis (post-treatment minus pre-treatment). A threshold of voxel-wise *P* < 0.005 uncorrected and cluster-level *P* < 0.05 false discovery rate (FDR) corrected will be applied to all analyses. Pearson’s correlation test will be conducted to investigate the changes between fMRI data and corresponding clinical data in each group.

## Discussion

“If acupuncture cannot achieve the purpose of treatment, moxibustion is more appropriate” is a classic exposition on the rational clinical application of acupuncture therapy and moxibustion therapy. This study is the first to investigate the central mechanism of acupuncture therapy and moxibustion therapy for patients with PDM. The results will deepen the understanding of the similarities and differences between acupuncture and moxibustion in clinical efficacy and their central mechanism.

### Rs-fMRI is an appropriate approach to explore the central mechanism of acupuncture and moxibustion

Since the mid-1990s, quantifying how acupuncture and moxibustion affect the activity of the central nervous system (CNS) with neuroimaging techniques has attracted increasing attention. With the advantages of non-invasive, non-radiation, and high temporal and spatial resolution, fMRI has emerged as the most widely used neuroimaging technique [33–35]. Our previous bibliometric study also demonstrated that 82.14% of the acupuncture-neuroimaging studies used fMRI to explore the cerebral responses to acupuncture stimulation [36]. Resting-state (rs)-fMRI, one of the main types of fMRI, refers to the spontaneous regulation activity of neurons in the relevant brain area of the brain obtained by MRI when subjects are awake and in the resting state without specific brain activity [37]. Compared with task-related fMRI, rs-fMRI helps to better elucidate the intrinsic and spontaneous neural activity of the brain. In recent years, a large number of rs-fMRI studies have been performed to unravel the mystery of brain regulation of acupuncture analgesia [38–40]. Taken together, rs-fMRI can be a useful tool to investigate the central mechanism of acupuncture and moxibustion.

### Central mechanism plays an important role in achieving the efficacy of both acupuncture and moxibustion

Accumulating evidences suggest that the response and integration of the central system to acupuncture stimulation are an important factor affecting the acupuncture effect [17]. The therapeutic effects of acupuncture depend on its dynamic reconfiguration of complex neural networks [41]. Our previous rs-fMRI study demonstrated that acupuncture could achieve treatment effects by modulating brain networks associated with the descending pain modulatory system in patients with PDM [42].

At the same time, neuroimaging studies on moxibustion have also gradually aroused the investigator’s interest. For example, the study has shown that the use of moxibustion can relieve pain via correcting abnormal brain changes in patients with visceral pain [18]. It suggested that information integration of moxibustion stimulation in the central system is the key link to achieve the effect of moxibustion. In addition, Bao et al. [19] compared the brain responses of acupuncture and moxibustion in Crohn’s disease and found that acupuncture regulated the homeostatic afferent processing network, while moxibustion mainly regulated the default mode network of the brain. These studies provide references and the possibility to explore the difference between acupuncture and moxibustion from the perspective of the central mechanism.

### Methodological quality control is crucial to ensure the repeatability of the results

Strict quality control is essential to improve the reliability of the study and should be used throughout the test. In this trial, quality control will be strengthened in three aspects. Firstly, when recruiting patients, we establish rigorous inclusion and exclusion criteria. In addition to demographic characteristics, other factors such as the usage of medication that may influence cerebral activities are also considered. Because PDM is often accompanied with anxiety and depression, SAS and SDS are used to assess the emotional state. Secondly, in order to ensure the curative effect, unified training will be conducted before the acupuncture and moxibustion operation. After the training is qualified, the two acupuncturists will follow the strict and standard operating procedures. Thirdly, baseline data will be collected on the first day of the menstrual cycle. Participants will be asked to maintain their regular lifestyle and avoid staying up late and ingesting alcohol and caffeine 24 h before the scan. Meanwhile, participants will be asked to follow uniform instructions, wear earplugs, close their eyes, and remain relaxed and awake during the scan.

Acupuncture and moxibustion are effective interventions in PDM treatment, but their central mechanism remains unclear. This trial is designed to investigate the differences between acupuncture and moxibustion in clinical efficacy and central mechanism through the following two aspects. On the one hand, the symptom improvement and cerebral responses to acupuncture, moxibustion, and waiting list will be compared, and on the other hand, the correlation between the cerebral activity changes and clinical variables’ improvement will also be analyzed.

### The strengths and limitations of this study

There are some strengths of this study as follows. Firstly, acupuncture and moxibustion, as two important parts of the external treatment of TCM, are widely used to treat various pain diseases, including PDM. Although previous studies have found that acupuncture and moxibustion can effectively relieve the clinical symptoms of patients with PDM [12, 13, 15], the difference in clinical efficacy and the underlying mechanism remains unclear. Therefore, this study will first investigate the potential similarities and differences between acupuncture and moxibustion in clinical efficacy and central mechanism for patients with PDM. Secondly, this study will observe structural and functional changes in the patients with PDM at baseline and at the end of the treatment. The application of a randomized controlled neuroimaging trial will provide objective and valid evidence on how acupuncture and moxibustion treatment relieves dysmenorrhea by modulating brain networks associated with pain and analgesia.

There are also some limitations in this study. Firstly, due to the particularity of acupuncture and moxibustion operation, the blind method cannot be achieved. Secondly, this study focused on the clinical symptoms and structural and functional changes in the patients with PDM before and after acupuncture or moxibustion treatment, so the follow-up period of patients is not investigated in this study.

### Trial status

This trial was registered at the Chinese Clinical Trial Registry (http://www.chictr.org.cn) on 27 February 2021 (registered number: ChiCTR2100043732, the protocol version number: V2.0). This study is currently in the recruitment stage. The first patient was enrolled on 1 March 2021. Recruitment will be approximately completed before 30 June 2022, and the trial is estimated to end in December 2022.

## Data Availability

The data and the relevant results in this study will be shared through scientific papers.

## Abbreviations

AE: Adverse events
ANOVA: Analysis of variance
BOLD-fMRI: Blood oxygenation level-dependent functional magnetic resonance imaging
CDUTCM: Chengdu University of Traditional Chinese Medicine
CMSS: COX menstrual symptom scale
CNS: Central nervous system
CRF: Case report form
3D-T1WI: Three-dimensional T1-weighted imaging
FDR: False discovery rate
fMRI: Functional magnetic resonance imaging
FOV: Field of view
NSAIDs: Nonsteroidal anti-inflammatory drugs
PCS: Pain catastrophizing scale
PDM: Primary dysmenorrhea
QoL: Quality of life
SAE: Serious adverse events
SAS: Self-rating anxiety scale
SDS: Self-rating depression scale
SF-36: 36-Item short form health Survey
TCM: Traditional Chinese medicine
TE: Echo time
TR: Repetition time
VAS: Visual analog scale

## References

[1] Rafique N, Al-Sheikh MH (2018). Prevalence of primary dysmenorrhea and its relationship with body mass index. J Obstet Gynaecol Res.

[2] Kennedy S (1997). Primary dysmenorrhoea. Lancet.

[3] Kho KA, Shields JK. Diagnosis and management of primary dysmenorrhea. JAMA. 2019. 10.1001/jama.2019.16921 [published Online First: 2019/12/20]. 10.1001/jama.2019.16921 31855238

[4] Proctor M, Farquhar C (2006). Diagnosis and management of dysmenorrhoea. BMJ.

[5] Liu P, Liu Y, Wang G (2017). Aberrant default mode network in patients with primary dysmenorrhea: a fMRI study. Brain Imaging Behav.

[6] Marjoribanks J, Ayeleke RO, Farquhar C, et al. Nonsteroidal anti-inflammatory drugs for dysmenorrhoea. *Cochrane Database Syst Rev*. 2015;(7):CD001751. 10.1002/14651858.CD001751.pub3 [published Online First: 2015/08/01]. 10.1002/14651858.CD001751.pub3 PMC6953236 26224322

[7] Osayande AS, Mehulic S (2014). Diagnosis and initial management of dysmenorrhea. Am Fam Physician.

[8] Davis AR, Westhoff CL (2001). Primary dysmenorrhea in adolescent girls and treatment with oral contraceptives. J Pediatr Adolesc Gynecol.

[9] Harel Z (2006). Dysmenorrhea in adolescents and young adults: etiology and management. J Pediatr Adolesc Gynecol.

[10] Wong CL, Farquhar C, Roberts H, et al. Oral contraceptive pill as treatment for primary dysmenorrhoea. *Cochrane Database Syst Rev*. 2009;(2):CD002120. 10.1002/14651858.CD002120.pub2 [published Online First: 2009/04/17]. 10.1002/14651858.CD002120.pub2 19370576

[11] Liu CZ, Xie JP, Wang LP (2011). Immediate analgesia effect of single point acupuncture in primary dysmenorrhea: a randomized controlled trial. Pain Med.

[12] Woo HL, Ji HR, Pak YK (2018). The efficacy and safety of acupuncture in women with primary dysmenorrhea: a systematic review and meta-analysis. Medicine (Baltimore).

[13] Yang M, Chen X, Bo L (2017). Moxibustion for pain relief in patients with primary dysmenorrhea: a randomized controlled trial. PLoS One.

[14] Armour M, Dahlen HG, Zhu X (2017). The role of treatment timing and mode of stimulation in the treatment of primary dysmenorrhea with acupuncture: an exploratory randomised controlled trial. PLoS One.

[15] Liu LY, Li XJ, Wei W (2020). Moxibustion for patients with primary dysmenorrhea at different intervention time points: a randomized controlled trial. J Pain Res.

[16] Yang J, Xiong J, Yuan T (2020). Effectiveness and safety of acupuncture and moxibustion for primary dysmenorrhea: an overview of systematic reviews and meta-analyses. Evid Based Complement Alternat Med.

[17] Han JS (2011). Acupuncture analgesia: areas of consensus and controversy. Pain.

[18] Zhu Y, Wu Z, Ma X (2014). Brain regions involved in moxibustion-induced analgesia in irritable bowel syndrome with diarrhea: a functional magnetic resonance imaging study. BMC Complement Altern Med.

[19] Bao C, Liu P, Liu H (2016). Different brain responses to electro-acupuncture and moxibustion treatment in patients with Crohn’s disease. Sci Rep.

[20] Chan AW, Tetzlaff JM, Altman DG (2013). SPIRIT 2013 statement: defining standard protocol items for clinical trials. Ann Intern Med.

[21] Burnett M, Lemyre M (2017). No. 345-primary dysmenorrhea consensus guideline. J Obstet Gynaecol Can.

[22] Desmond JE, Glover GH (2002). Estimating sample size in functional MRI (fMRI) neuroimaging studies: statistical power analyses. J Neurosci Methods.

[23] Hayasaka S, Peiffer AM, Hugenschmidt CE (2007). Power and sample size calculation for neuroimaging studies by non-central random field theory. Neuroimage.

[24] Yu S, Yang J, Ren Y (2015). Characteristics of acupoints selection of moxibustion for primary dysmenorrhea based on data mining technology. Zhongguo Zhen Jiu.

[25] Yu S, Yang J, Yang M (2015). Application of acupoints and meridians for the treatment of primary dysmenorrhea: a data mining-based literature study. Evid Based Complement Alternat Med.

[26] Cox DJ, Meyer RG (1978). Behavioral treatment parameters with primary dysmenorrhea. J Behav Med.

[27] Price DD, McGrath PA, Rafii A (1983). The validation of visual analogue scales as ratio scale measures for chronic and experimental pain. Pain.

[28] Sullivan MJLBS (1995). The pain catastrophizing scale: development and validation. Psychol Assess.

[29] Quartana PJ, Campbell CM, Edwards RR (2009). Pain catastrophizing: a critical review. Expert Rev Neurother.

[30] Zung WW (1971). A rating instrument for anxiety disorders. Psychosomatics.

[31] Zung WW, Richards CB, Short MJ (1965). Self-rating depression scale in an outpatient clinic. Further validation of the SDS. Arch Gen Psychiatry.

[32] Ware JE, Sherbourne CD (1992). The MOS 36-item Short-Form Health Survey (SF-36). I. Conceptual framework and item selection. Med Care.

[33] Huang W, Pach D, Napadow V (2012). Characterizing acupuncture stimuli using brain imaging with FMRI--a systematic review and meta-analysis of the literature. PLoS One.

[34] He T, Zhu W, Du SQ (2015). Neural mechanisms of acupuncture as revealed by fMRI studies. Auton Neurosci.

[35] Dhond RP, Kettner N, Napadow V (2007). Neuroimaging acupuncture effects in the human brain. J Altern Complement Med.

[36] Qiu K, Jing M, Sun R (2016). The status of the quality control in acupuncture-neuroimaging studies. Evid Based Complement Alternat Med.

[37] Greicius MD, Krasnow B, Reiss AL (2003). Functional connectivity in the resting brain: a network analysis of the default mode hypothesis. Proc Natl Acad Sci U S A.

[38] Yu S, Ortiz A, Gollub RL, et al. Acupuncture treatment modulates the connectivity of key regions of the descending pain modulation and reward systems in patients with chronic low back pain. *J Clin Med*. 2020;9(6). 10.3390/jcm9061719 [published Online First: 2020/06/07]. 10.3390/jcm9061719 PMC7356178 32503194

[39] Li Z, Lan L, Zeng F (2017). The altered right frontoparietal network functional connectivity in migraine and the modulation effect of treatment. Cephalalgia.

[40] Chen X, Spaeth RB, Freeman SG (2015). The modulation effect of longitudinal acupuncture on resting state functional connectivity in knee osteoarthritis patients. Mol Pain.

[41] Qin W, Bai L, Dai J (2011). The temporal-spatial encoding of acupuncture effects in the brain. Mol Pain.

[42] Yu S, Xie M, Liu S (2020). Resting-state functional connectivity patterns predict acupuncture treatment response in primary dysmenorrhea. Front Neurosci.

